# Genetic Variations of ferroportin-1(FPN1-8CG), TMPRSS6 (rs855791) and Hemojuvelin (I222N and G320V) Among a Cohort of Egyptian β-Thalassemia Major Patients

**DOI:** 10.1007/s12288-022-01580-8

**Published:** 2022-11-01

**Authors:** Nesrine El-Gharbawi, Iman Shaheen, Mona Hamdy, Somaya Elgawhary, Mohamed Samir, Baher Matta Hanna, Eman Yousief Ali, Eman Ahmed Youssef

**Affiliations:** 1 Clinical Pathology Department, Cairo University, Cairo, Egypt; 2 Pediatric Hematology, Department of pediatrics, Cairo University, Cairo, Egypt; 3 Clinical Pathology Department, FayoumUniversity, Fayoum, Egypt; 4 Pediatric Cardiology, Department of Pediatrics, Cairo University, Cairo, Egypt; 5 Lecturer of Hematopathology, Department of Clinical and chemical pathology, Cairo University, Cairo, Egypt; 6 Department of Clinical and Chemical Pathology, Faculty of Medicine, Cairo University, 11562 Cairo, Egypt

**Keywords:** β-TM, FPN 1, HJV, TMPRSS6

## Abstract

Iron overload remains a major cause of morbidity and mortality among β-thalassemia major (β-TM) patients. Iron regulatory proteins and their genetic variants together with changes in hepcidin levels in thalassemic patients could affect the disease manifestations. This work aimed to study genetic variations of ferroportin-1 (FPN1-8CG), Transmembrane Serine Protease 6 (TMPRSS6 rs855791) and hemojuvelin (HJV I222N and G320V) genes within a cohort of 97 β-TM Egyptian patients by Polymerase chain reaction Restriction Fragment Length Polymorphism (PCR-RFLP) in comparison to fifty normal control subjects. Among β-TM patients; the CG variant of FPN1 was significantly higher, while the TT and TC variants of TMPRSS6 were significantly lower in comparison to controls. Liver Iron Concentration (LIC) was significantly higher among β-TM patients harboring the FPN1 (GG) genotype and we found that FPN1gene mutation acts as independent predictor of MRI LIC (p = 0.011), Pulmonary artery pressure (PAP) was significantly higher in patients harboring the mutant FPN1 (GG and CG) genotypes (p value 0.04). β-TM patients having the HJV I222N (AA) genotype were having significantly higher cardiac iron overload (p value = 0.026). The studied genetic variants of iron regulatory proteins could alter the manifestations of iron overload thus resulting in different clinical phenotypes of thalassemic patients, these findings need to be confirmed by larger cohorts of patients with longer follow-up periods.

**Supplementary Information:**

The online version contains supplementary material available at 10.1007/s12288-022-01580-8.

## Introduction

β-thalassemia major (β-TM) is one of the most common human genetic disorders in the world, which is caused by various mutations in the β globin gene leading to imbalanced globin- chain synthesis, defective hemoglobin production and hemolytic anemia 1.

The combination of regular blood transfusions and chelation therapy has dramatically increased the life expectancy of thalassemia. On the other hand, frequent blood transfusion has also led to iron overload with many complications including, cardiovascular problems 2, 3.

Normally iron is tightly regulated by hepcidin, a peptide hormone influenced by iron levels in a complex process which includes other proteins such as; ferroportin-1 (FPN1), hemojuvelin (HJV) Transmembrane Serine Protease 6 (TMPRSS6) and others. Polymorphisms of genes coding for such proteins are linked to iron associated disorders ranging from iron deficiency anemia to hereditary hemochromatosis 4.

Hepcidin production is controlled by suppressive effects of erythropoiesis and stimulatory effects of iron overload. Regularly transfused patients should not experience significant reduction in serum hepcidin levels. Following transfusion, ineffective erythropoiesis is partially reduced resulting in an increase in serum hepcidin. Consequently,

hepcidin levels would reflect the quality of management of ineffective erythropoiesis in patients with β-thalassemia 5.

Hepcidin is confirmed to be down-regulated in Egyptian β-TM 6, more researches are needed to investigate the genetic interaction of the proteins involved in the maintenance of Iron balance in the tissue and how they together with such low hepcidin levels influence the cellular Iron levels 7.

Up to our best knowledge this is the first study aiming to study the prevalence of ferroportin-1 (FPN1-8CG), TMPRSS6 (rs855791) and HJV (I222N & G320V) gene polymorphisms among β-TM patients and to investigate their possible effect on the disease manifestations, in an attempt to identify high risk patients who could benefit from enhancing iron chelation therapy or new therapeutic modalities to reduce the overall morbidity and mortality.

## Methods

### Study Group

Our study included 97 previously diagnosed β-TM patients (based on their clinical and laboratory results) ; 56 males (57.7%) and 41 females (42.3%) with a mean age of 18.6 ± 9.3 years, patients were registered as follow-up cases at the Hematology Clinic of the Children Hospital of Cairo University. In addition, 50 age and sex matched normal subjects were included as a control group.

The study design was approved by the Scientific Research Committee of Clinical and Chemical Pathology and Pediatrics Departments of Faculty of Medicine, Cairo University. Formal consents were obtained from the participants or their guardians who agreed to participate in the study. All procedures followed were in accordance with the ethical standards of the responsible committee on human experimentation (institutional and national) and with the Helsinki Declaration of 1975, as revised in 2008.

All patients included in the study were subjected to thorough clinical evaluation. Laboratory investigations were done including; complete blood picture, reticulocytic count, Aspartate and alanine amino-transferases (AST and ALT), Hepatitis C virus (HCV) antibodies were tested using enzyme immunoassay (EIA), and serum ferritin level using micro particle enzyme immunoassay (MEIA).

### Radiological Investigations

**Echocardiography**: Two-dimensional, pulsed-wave, continuous-wave and color-flow Doppler echocardiography was performed. Using the M-Mode, left atrial and aortic dimensions were assessed, in long axis and parasternal views, the following were measured: right ventricular end diastolic diameter (RVEDD), left ventricular end-diastolic diameter (LVEDD), left ventricular end-systolic diameter (LVESD), inter-ventricular septum (IVS) and posterior wall thickness (PWT). In addition to those, calculation of fraction shortening (FS%) and ejection fraction (EF%) were used as an indication of LV systolic function according to the recommendations of the American Society of Echocardiography.

#### Magnetic Resonance Imaging (MRI)

Cardiac and Hepatic siderosis were assessed using SDPA R2-MRI (FerriScan®), cardio-vascular magnetic resonance (CMR) relaxation time T* was measured in milliseconds (ms), while liver iron concentration (LIC) was expressed in milligram per gram dry tissue (mg/ g). **C**ardiac T2* above 20 ms **and** LIC below 3 mg/ g were considered normal 8.

### 2.3 Genomic DNA Analysis (Genotypic Analysis)

Detection of FPN-1(-8CG), TMPRSS6 (rs855791) and HJV (I222N & G320V) gene polymorphisms was done by PCR- RFLP analysis.

Two milliliters of venous blood were collected on EDTA vacutainer tubes. Samples were stored at -20° C till DNA extraction which was performed using the Gene JET Blood Genomic DNA Purification Kit (Thermo Scientific) # K0721.

This was followed by amplification of the extracted DNA for detection of different polymorphisms using PCR technique in separate reactions. Restriction of the PCR amplification products (amplicon) by specific restriction enzyme for each polymorphism was done, followed by visualization of the PCR-restriction fragments using ethidium bromide stained agarose gel electrophoresis under UV light against a standard DNA ladder. The results were documented using a digital camera. The designed primers, reaction conditions, end product amplicon size, restriction enzymes used as well as lengths of generated fragments for each polymorphism were summarized in Table 1. For quality control, genotyping of the studied polymorphisms was repeated for 30 randomly chosen samples of the patients and controls and interpreted blindly by two different observers. The results were concomitant with the initial results.

**Table 1:** PCR conditions of the studied polymorphisms

SNP	Primer sequence	Reaction conditions	product size (bp)	Restriction Enzyme	Fragment Length
FPN1 -8CG	Forward : 5’CCAGTTCCTTGCACTCCTG-3’ Reverse : 5’CATCCTCTCTGGCGGTTG-3’	→→5’94^o^C 30(30’’94^o^C + 30’’60 ^o^C+ 60’’72^o^C) 5’72^o^C	129	*BstU I*	CC;129 GG; 85, 44 CG;129, 85, 44
TMPRSS6 rs855791	Forward : 5’TAGAGAACAGGGGCTCCAGG 3’ Reverse : 5’ATGTGGGCAGCATCCTTTC 3’	→→5’94^o^C 30(45’’95^o^C + 45’’64^o^C + 45’’72^o^C) 5’72^o^C	249	*Stu I*	CC;249 TT;125 TC; 249, 125
HJV I222N	Forward : 5’TAGTCCTGCATCTCTACTTGG 3’ Reverse : 5’AGCTGCCACGGTAAAGTTGG3’	→→5’94^o^C 30(30’’94^o^C + 30’’60^o^C + 30’’72^o^C) 5’72^o^C	581	*Bcc I*	TT; 233,159,120,69 AA; 581 TA; 581,233,159,120,69
HJV G320V	Forward : 5’TAGTCCTGCATCTCTACTTGG 3’ Reverse : 5’AGCTGCCACGGTAAAGTTGG3’	→→5’94^o^C 30(30’’94^o^C + 30’’60^o^C + 30’’72^o^C) 5’72^o^C	581	*Ban I*	GG; 409,172 TT; 581 TG; 581,409,172

### Statistical Analysis

Statistical package for the Social Sciences (SPSS) version 25 (IBM Corp., Armonk, NY, USA) was used for Coding, entering data and statistical analysis. Data were summarized using mean, standard deviation, median, minimum and maximum in quantitative data and using frequency (count) and relative frequency (percentage) for categorical data. Genotype and allele frequencies were compared between the disease and the control groups. Comparisons between quantitative variables were done using the non-parametric Kruskal-Wallis and Mann-Whitney tests. For comparing categorical data, Chi square (χ2) test was performed. Exact test was used instead when the expected frequency is less than 5. P-values less than 0.05 were considered as statistically significant.

Multivariate linear regression analysis was done to examine possible confounders in significant relations 9 e.g., patient age, age of onset of transfusion, transfusion duration, chelation therapy duration, chelation therapy compliance and serum ferritin level.

## Results

The demographic, laboratory, clinical and radiological data of the study group are provided as a supplementary table.

In Our patients’ group, the mean age of the first transfusion was 15.7 ± 12.07 months, the mean interval between transfusions was 25.2 ± 9.56 days and the mean time of their last transfusion was 21.5 ± 17.04days.

The MRI study of heart and liver showed that the mean cardiac T2* was 29.69 ± 14.66 ms. and the mean LIC was 16.4 ± 8.40 mg/g .

The prevalence of the 4 studied polymorphisms among the cases and control subjects are listed in Table 2. The heterozygous FPN1 (CG) genotype was significantly higher among β-TM patients than in controls (p = 0.025). The homozygous (TT) and heterozygous (TC) genotypes of TMPRSS6 were significantly lower among β-TM ( p = 0.042, 0.049 respectively ).

**Table 2:** Genotyping of FPN1, TMPRSS6, HJV I222N and HJV 320 polymorphisms in patients and controls:

			Cases				Controls			
			**Count**	**%**			**Count**		**%**	**P value**
**FPN I**		**CC**	61	62.9%			40		80.0%	Reference
		**GG**	3	3.1%			2		4.0%	0.986
		**CG**	33	34.0%			8		16.0%	**0.025**
		**CG + GG**	36	37.1%			10		20.0%	**0.037**
**TMPRSS6**		**CC**	16	16.5%			2		4.0%	Reference
		**TT**	40	41.2%			25		50.0%	**0.042**
		**TC**	41	42.3%			23		46.0%	**0.049**
		**TC + TT**	81	83.5%			48		96.0%	**0.044**
**HJV I222N**		**TT**	92	94.8%			47		94.0%	Reference
		**AA**	1	1.0%			1		2.0%	0.638
		**TA**	4	4.1%			2		4.0%	0.981
		**TA + AA**	5	5.2%			3		6.0%	0.831
**HJV 320**	**GG**		94		96.9%	48		96.0%		Reference
	**GT**		3		3.1%	2		4.0%		0.774

**Fig. 1:**
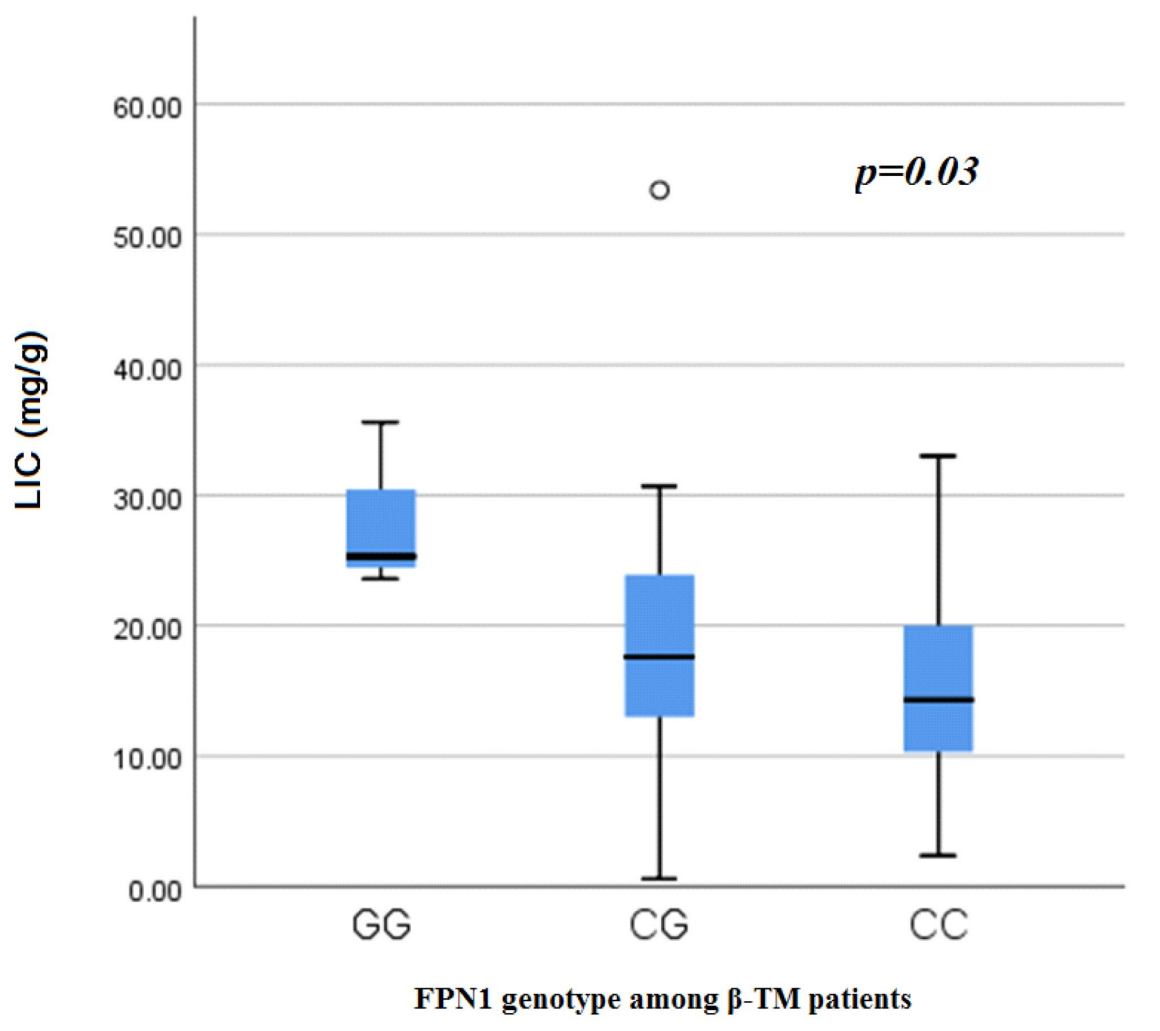
Liver Iron concentration among β-TM patients in accordance to FPN1genotypes.

MRI LIC was significantly higher in patients harboring the homomutant FPN1 (G/G) genotype than those with the wild type (p = 0.03) **(**Fig. 1**)**. This significance was maintained after multivariate linear regression analysis (p = 0.011) **(**Table 3**)**.

**Table 3:** Multivariate linear regression analysis between MRI LIC as dependent numerical variable and mutant FPN1 (G/G) genotype as independent variable adjusted for patient age, age of onset of transfusion, transfusion duration, chelation therapy duration, chelation therapy compliance and serum ferritin

Model		Unstandardized Coefficients		Standardized Coefficients	t	P value	95% Confidence Interval for B	
		**B**	**Std. Error**	**Beta**			**Lower Bound**	**Upper Bound**
MRI LIC	**FPN I (wild/mutant)**	3.856	1.492	0.221	2.585	0.011	0.891	6.821
	**AGE year**	0.229	0.128	0.247	1.790	0.077	− 0.025	0.483
	**AGE OF 1 ST TRANSFUSION (month)**	− 0.201	0.064	− 0.288	-3.128	0.002	− 0.329	− 0.073
	**TRANSFUSION duration (years)**	− 0.093	0.066	− 0.140	-1.404	0.164	− 0.225	0.039
	**CHELATION THERAPY DURATION (YEAR)**	0.122	0.140	0.109	0.873	0.385	− 0.156	0.401
	**COMPLIANCE (CHELATION THERAPY)**	2.187	1.759	0.114	1.244	0.217	-1.309	5.683
	**FERRITIN (mg/dl)**	0.001	0.000	0.454	4.945	< 0.001	0.001	0.002

**Fig. 2:**
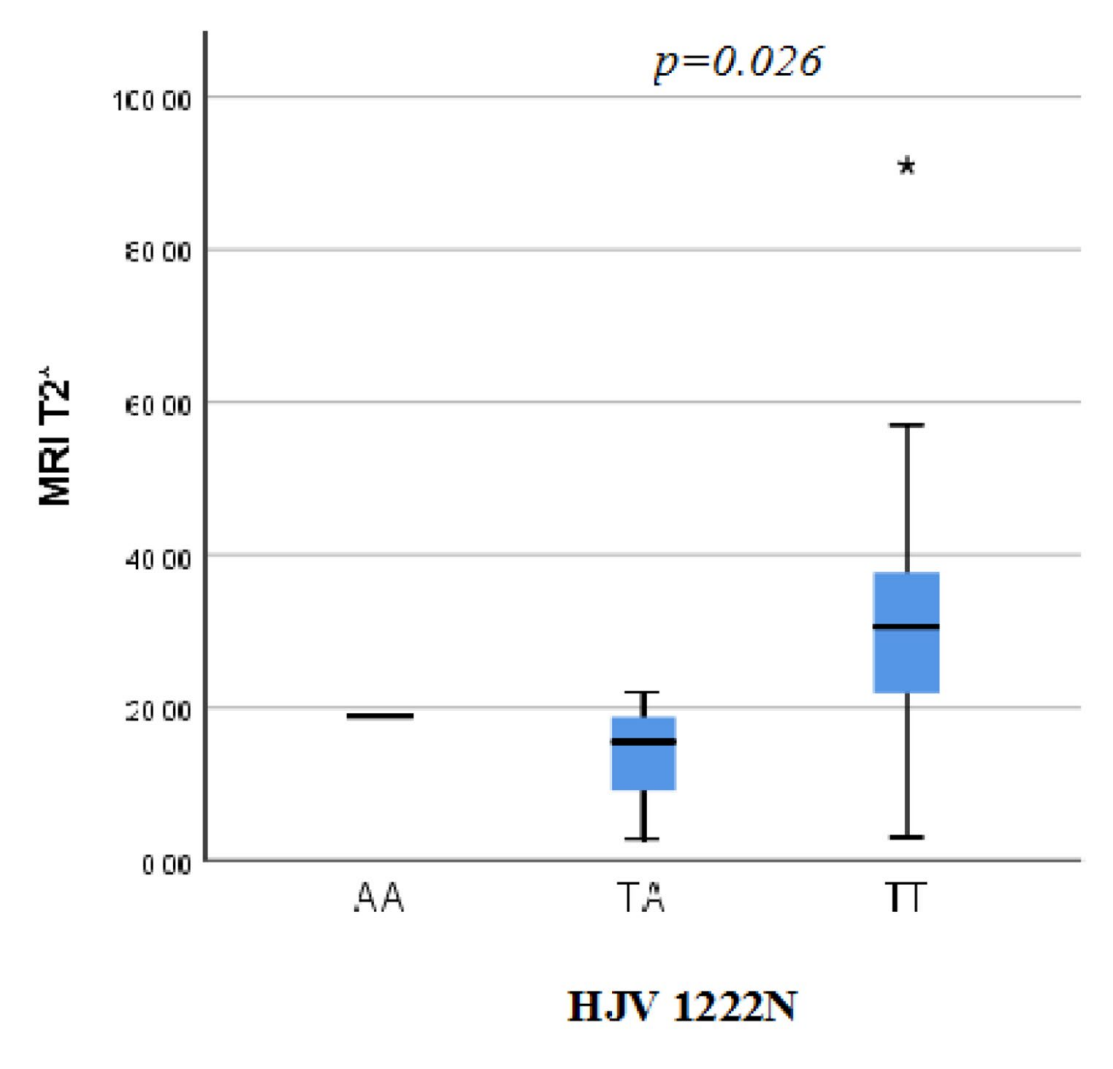
Cardiac MRI T2* of the β-TM patients in accordance to HJV 1222 N genotypes

Also, Pulmonary artery pressure (PAP) was significantly higher in patients harboring the mutant FPN1 genotypes (GG + GC) as the mean of PAP among those patients was 30.5 ± 6.1mmHg on comparison to the patients harboring the wild (CC) gentotype as their PAP was 26 ± 5.13mmHg (p = 0.04). MRI T2* was significantly lower among homomutant HJV I222N SNP (p = 0.026) (Fig. 2**)** LVPWT was significantly higher in patients harboring the TA + AA variant of HJV1222N genotypes (10.4 ± 1.3 mm) than those with the wild TT genotype (8.7 ± 1.8 mm) (p = 0.030). Multivariate linear regression analysis was done to examine the fore mentioned significant relations however the significance was not maintained after the statistical adjustment of other variables as possible confounders.

No statistically significant difference was noticed between patients harboring the wild (TT), heteromutant (TC) or homomutant(CC) genotypes of TMPRSS6 SNP as regarding all clinical, laboratory and radiological data.

No statistically significant difference was noticed between patients harboring the wild or the mutant genotypes of HJV G320V SNP as regarding all clinical, laboratory and radiological data.

## Discussion

Iron overload remains to be a major complication among the frequently transfused β-TM patients, especially if patients are not compliant to the iron chelation therapy. The demographic data of the present study illustrates a high burden of Iron overload in Egyptian β-TM patients as the mean serum ferritin was 4566.6 ± 3287.3ng/ml, this high value was a.

The local Iron tissue accumulation assessed by MRI of the liver and heart showed that the mean LIC of the patient’s group was 16.4 ± 8.40 mg/g, while the mean patient’s cardiac T2* was 29.69 ± 14.66 ms. These findings highlight the need to improve the implementation of patients follow up and education programs among the Egyptian β-TM to fulfill adequate patient compliance to iron chelation therapy, as unfortunately 72 patients (74.2%) of our β-TM patients were non-compliant to the iron chelation therapy.

Another challenging consequence of the recurrent blood transfusion that faces the health care system is the prevalence of hepatitis C viral (HCV) infection. In our study, 28 patients (29%) were positive for HCV infection; this is lower than the incidence reported among Egyptian β-TM in 2016 (37.11%) 10 which in turn is less than the incidence reported in 2012(69%) 11.

In 2008, Egypt had the highest burden of HCV infection worldwide. In 2015, the seroprevalence of HCV infection in Egypt has declined to 6.3% 12 we believe that this would be reflected as a lower HCV incidence among β thalassemia patients in future studies.

To the best of our knowledge, the prevalence of feroportin-1(-8CG), TMPRSS6 (rs855791) and HJV (I222N & G320V) gene polymorphisms in β-TM patients and the study of their possible relation to the development of iron overload was not previously studied but the effect of those genotypic variations was investigated in other conditions of altered iron metabolism.

This study revealed that heterozygous FPN1 C/G genotype was statistically significantly higher in patients (34%) when compared to the controls (16%), the wild C/C genotype was detected in 80% of the control subject and 62.9% of the cases, while 2 control subjects and 3 cases harbored the homozygous GG genotype with no statistical difference.

No significant relation could be detected between the FPN1 -8CG polymorphism and the level of serum ferritin denying the possibility of its contribution to increasing the soluble systemic iron stores however local liver iron reflected by the MRI LIC was significantly higher in patients harboring the (GG) FPN1 genotype when compared to those with the wild type (CC) genotype (p value = 0.03) suggesting the possible role of the FPN1 -8CG polyomorphism in enhancing the local iron overload within the liver. After statistical adjustment of other variables, we found that FPN1gene mutation acts as independent predictor of MRI LIC (p = 0.011).

Also, PAP was significantly higher in patients harboring the (GG + GC) FPN1 genotype in comparison to those with the wild (CC) genotype (p value = 0.048) suggesting the role of this mutation in producing local effects on the cardio vascular functions.

Ferroportin is the only known cellular iron exporter. It is regulated at a post-translational level by the main iron regulatory peptide; hepcidin. Decreased ferroportin expression would reduce external iron export and maintain intracellular iron 13. The FPN1 -8CG position is extremely close to the iron responsive element (IRE) of the gene, thus potentially interfering with FPN1 expression 14.

Despite being not previously studied among β-TM patients, FPN1 gene mutations have been investigated in other conditions of tissue iron overload. Castiglione et al., in 2015 studied the possible role of FPN1 − 8CG Polymorphism in modifying the iron homeostasis at the local level where they found that the homozygous genotype FPN1 − 8GG was significantly associated with increased risk of developing sudden sensory-neural hearing loss. It was assumed that increased levels of Fe2 + inhibit the intracellular production of nitrous oxide, a fundamental element in the regulation of local microcirculation 15.

Gemmati et al., 2009 studied the FPN − 8CG SNP and reported that patients harboring the (GG) genotype had a significant 5 folds increase in risk of acquiring chronic venous ulcer, this could be attributed to alteration of the macrophage membrane interfering with the efflux of iron from inside the cell with consecutive increase in oxidative stress 14.

Another study for the same SNP was conducted in Multiple sclerosis (MS) patients and concluded that there was a 4 folds increased risk of MS associated with the FPN1 -8GG homozygous genotype. In addition, disease progression and severity gradually increased as the number of the G alleles increased 16.

This however doesn’t agree with the results obtained by Tisato et al., were it was found that FPN1 -8G allele was under represented in vascular dementia patients although iron-driven oxidative stress is a key recognized physiopathological contributor for the disease, yielding a significant 1.5 fold risk reduction 17.

Regarding the TMPRSS6 genotypes, in this study, the homozygous TMPRSS6 (TT) genotype was statistically significantly lower in patients (41.2%) when compared to the controls (50%). The iron lowering allele, T allele, in homozygous state (TT) or heterozygous state (TC) was significantly lower in β-TM patients when compared to the control group (p = 0.042 and 0.049 respectively). This could point to a role of this genotype in iron overload pathogenesis and expression.

A study for the effect of the TMPRSS6 rs855791 polymorphism and consequent increase in hepcidin release concluded that TMPRSS6 polymorphism is likely a modifier of hereditary chromatosis (HH) expression 18. Also, a systematic review performed by Gichohi-Wainaina et al. in 2015 with meta-analysis done on TMPRSS6 loci identified in cohorts of differing ethnicity, they concluded that the T allele of rs855791 was associated with lower Hb and ferritin concentrations in all populations. This allele was also associated with increased serum transferrin receptor and transferrin concentrations 19.

A study carried out on the TMPRSS6 rs855791 polymorphism in the Egyptian population in 2018 showed a significant increase in frequency of mutations (TT and TC) in the counterpart disease of iron deficiency anemia patients compared to the normal group 20.

Hemojuvelin (HJV) is another protein that plays a crucial role in the regulation of hepcidin 21.

In the current study, no significant difference was found between the patients and control groups regarding the HJV 1222 N and HJV G320V genotypes frequencies (p = 0.831 and 0.774 respectively).

The present work showed significantly lower T2 among β-TM patients harboring the HJV 1222 N mutant variants reflecting higher cardiac iron concentration (p = 0.026). However LIC did not differ significantly among β-TM patients having various HJV 1222 N SNP genotypes.

Moreover, LVPWT was significantly higher in patients harboring the variant HJV1222N genotypes compared to those with the wild type (p value = 0.030) suggesting a higher degree of cardiac impairment in this group.

The current study revealed that 3 β-TM patients (3.1%) and 2 control subjects (4%) harbored the mutant allele for HJV G320V SNP.

Some studies have reported that the HJV gene mutations are uncommon.^22^, ^23^. The relative higher prevalence of those rare genotypes in the Egyptian population could be attributed to the higher incidence of consanguinity in marriage 24.

Previous studies have reported that HJV gene polymorphisms act as genetic modifiers in the hereditary hemochromatosis (HH) phenotype. A study in 2004 reported that association of heterozygous mutations in the HFE and HJV mutations could lead, at least in some cases, to an adult-onset form of primary iron overload 25 however this does not agree with the results obtained by a gene sequencing study conducted in 2010 26, also Alte`s et al. in 2009 found a minor impact of these mutations in the development of HFE-related hemochromatosis 27.

In conclusion this study identified that FPN1gene mutation could be considered as a reliable independent predictor of MRI LIC (p = 0.011), in addition the PAP was significantly higher in patients harboring the mutant allele of the same SNP.

Although this study results need to be confirmed by larger cohorts of patients with longer follow-up periods, our results suggest that established genetic risk factors in β-TM might cause different clinical phenotypes and thus make patients differently suited to iron over load management strategies.

## Electronic Supplementary Material

Below is the link to the electronic supplementary material.

Supplementary Material 1
