# Supplementary material for: Genetic Variations of ferroportin-1(FPN1-8CG), TMPRSS6 (rs855791) and Hemojuvelin (I222N and G320V) Among a Cohort of Egyptian β-Thalassemia Major Patients
Source: Indian J Hematol Blood Transfus. 2022 Nov 1;39(2):258–65. doi: 10.1007/s12288-022-01580-8 (PMC10064347; doi:10.1007/s12288-022-01580-8)
Supplement: Supplementary file 1 — Supplementary Material 1 [file 12288_2022_1580_MOESM1_ESM.docx]

Supplementary data

**Demographic, clinical and Laboratory data of the B Thalassemia patients**

| **Parameter** | **Range** | **Mean + SD in the patients group** | **Median** |
| --- | --- | --- | --- |
| Age (years) | 8-52 | 18.6 ± 9.3 | 16.0 |
| Hemoglobin level (gm/dL) | 6-10.5 | 8.47 ± 5.04 | 8.00 |
| Mean Serum ferritin (ng/ml) | 214 – 13623 | 4566.6 ± 3287.3 | 3800.00 |
| AST^$^ (IU/L) | 6 – 380 | 59.42 +49.3 | 45.00 |
| ALT^$^ (IU/L) | 1 to 306 | 58.97± 50.5 | 46.00 |
| Duration of chelation therapy (years) | 0 – 35 | 12.3 ± 7.64 | 10 |
| LVEDD^*^ (mm) | 35.00- 62.00 | 48.39+6.85 | 47.00 |
| LVESD **(mm) | 11.00 – 44.00 | 30.89+5.38 | 31.00 |
| EF%*** | 46.00- 80.00 | 67.21+6.22 | 67.00 |
| FS%^#^ | 24.00 – 49.00 | 37.22+5.02 | 37.00 |
| IVST ^##^(cm) | 0.6 – 1.40 | 0.9+ 0.23 | 0.90 |
| LVPWT^###^ (cm) | 0.50 - 1.44 | 0.87+ 0.18 | 0.90 |
| E/A | 0.77 – 3.30 | 1.68+0.48 | 1.65 |
| PAP ^$$$^(mmHg) | 19.00 – 41.00 | 28.61+5.82 | 28.50 |
| **Parameter** | | **Number among patients group (Percentage)** | |
| Sex | | 56 males (57.7 %)  41 females (42.3%) | |
| Splenomegaly | | 26 patients (26.8%) | |
| Splenectomy | | 71 patients (74.2%) | |
| Hepatomegaly | | 33 patients (34.7%) | |
| Diabetes Mellitus | | 6 patients (6.8%) | |
| Recipients of Deferoxamine | | 76 patients (78.4%) | |
| Recipients of Deferiprone | | 48 patients (78.4%) | |
| Recipients of Deferasirox | | 89 patients (91.8%) | |
| Patients compliant to chelation therapy | | 25 patients (25.8%) | |
| HCV antibodies positive patients | | 28 patients (29.8%) | |

$ aspartate aminotransferase $$ alanine transaminase*Left ventricular end diastolic diameter**Left ventricular end systolic diameter*** Ejection fraction #Fraction shortening ##Inter Ventricular septal thickness. ###Left ventricular posterior wall thickness $$$Pulmonary artery pressure
